# Peer Review of “Development of a Conversational Artificial Intelligence–Based Web Application for Medical Consultations: Prototype Study”

**DOI:** 10.2196/84443

**Published:** 2025-10-15

**Authors:** Anonymous

**Keywords:** artificial intelligence, ChatGPT, chatbots, conversational agent, machine learning

*This is the peer-review report for “Development of a Conversational Artificial Intelligence–Based Web Application for Medical Consultations: Prototype Study.”*

## Round 1 Review

### General Comments

This paper [1] presents a novel conversational artificial intelligence (AI) that is built on top of several models capable of detecting various health conditions. The research itself is interesting, relevant, and carried out well. I look forward to seeing the revised work!

### Specific Comments

#### Major Comments

1. Please strongly consider rechecking the grammar for the paper as I found several mistakes and inconsistencies in just the Abstract alone. Grammar issues have made it difficult to follow several lines of thought throughout the paper and have lowered its overall quality. Furthermore, the style of writing is more conversational than it is formal and academic in several cases. This is my main reason for rejecting the paper. Please address these issues and then resubmit.
2. Because the experience is integrated into a chatbot, indicating that providing an interactive user experience was a goal of this work, it would be good to also conduct some user research to assess the usability of the system and participants’ impressions of it.
3. It is important to also include a section discussing the potential dangers and ethical implications of deploying such a chatbot in the real world given the sensitive context and its critical implications.

#### Minor Comments

1. I find the opening sentence of the Abstract and Introduction (“Artificial intelligence (AI) evolved in trends. Currently, the trend is conversational artificial intelligence (CAI).”) to be problematic. It is unclear what the statement “AI evolved in trends” means and therefore it is difficult to evaluate its accuracy. Furthermore, it would be more apt to say that the trend now is generative AI, which translates to large language models, that is fuelling conversational AI. It is also worth noting that conversational AI is not just focused on text-based interactions, but also includes voice modalities.

## Round 2 Review

### General Comments

Thank you to the authors for reading our comments and revising the paper. Many of our previous comments have been addressed; however, I still believe the writing style, grammar, and language of the paper need significant work before this can be published.

## Abbreviations

AI: artificial intelligence

## References

[1] Pires JG (2025). Development of a Conversational Artificial Intelligence–Based Web Application for Medical Consultations: Prototype Study. JMIRx Med.

